# Systematic review on the current state of disaster preparation Simulation Exercises (SimEx)

**DOI:** 10.1186/s12873-023-00824-8

**Published:** 2023-05-24

**Authors:** Syed Sarosh Mahdi, Hafsa Abrar Jafri, Raheel Allana, Gopi Battineni, Mariam Khawaja, Syeda Sakina, Daniyal Agha, Kiran Rehman, Francesco Amenta

**Affiliations:** 1 Jinnah Medical and Dental College, Department of Community Dentistry, Sohail University, Karachi, Pakistan; 2 Division of Clinical Oral Health Sciences, School of Dentistry, International Medical University, Kuala Lumpur, Malaysia; 3 Department of Paediatrics & Child Health, Aga Khan University Karachi, Karachi, 74800 Pakistan; 4 Clinical research centre, School of Medicinal and Health Products Sciences, University of Camerino, Camerino, 62032 Italy; 5 Sociology department, University of North Texas, Denton, TX76203 USA; 6 Division of Restorative Dentistry, International Medical University, Kuala Lumpur, Malaysia

**Keywords:** Disaster preparedness, Emergency response, SimEx, Mass Casualty Exercise (MCI), Disaster drills

## Abstract

**Introduction:**

The simulation exercise (SimEx) simulates an emergency in which an elaboration or description of the response is applied. The purpose of these exercises is to validate and improve plans, procedures, and systems for responding to all hazards. The purpose of this study was to review disaster preparation exercises conducted by various national, non-government, and academic institutions.

**Methodology:**

Several databases, including PubMed (Medline), Cumulative Index to Nursing and Allied Health Literature (CINAHL), BioMed Central, and Google Scholar, were used to review the literature. Information was retrieved using Medical Subject Headings (MeSH) and documents were selected according to Preferred Reporting Items for Systematic Reviews and Meta-Analyses (PRISMA). To assess the quality of the selected articles, the Newcastle-Ottawa Scale (NOS) technique was utilized.

**Results:**

A total of 29 papers were selected for final review based on PRISMA guidelines and the NOS quality assessment. Studies have shown that many forms of SimEx commonly used in disaster management including tabletop exercises, functional exercises, and full-scale exercises have their benefits and limitations. There is no doubt that SimEx is an excellent tool for improving disaster planning and response. It is still necessary to give SimEx programs a more rigorous evaluation and to standardize the processes more thoroughly.

**Conclusions:**

Drills and training can be improved for disaster management, which will enable medical professionals to face the challenges of disaster management in the 21st century.

## Introduction

The increase in population, resource scarcity, and escalating conflicts has increased both human-made and natural disasters around the world. A medical response team that is efficient and effective is crucial to improving the outcome of disaster response. It is becoming increasingly apparent that disaster medicine is progressing both theoretically and practically over the years as more research is conducted [1]. There has been an increase in research on disaster preparedness in public health [2].

World Health Organization (WHO) has stated that emergency relief supplies, preparedness, and prevention measures are equally important [3]. In a situation like this, disaster management and emergency preparedness are of the utmost importance. However, current disaster management training and education consist mainly of lectures and hospital drills. [4]. It is important to enhance disaster readiness through health readiness and drills, which prevents psychological, economic, physical, moral, and financial harm to society. There needs to be a uniform way of educating emergency medicine residents and other health professionals about disaster management.

Pandemics are worldwide health emergencies that take some countries off guard, exposing serious flaws in their ability to deal with similar calamities. As a result of the recent COVID-19 pandemic, the worlds mass emergency response protocol has once again been debated at the federal, state, and local levels. COVID-19 has highlighted inadequacies in emergency planning among governments and international partners. Simulation Exercises (SimEx) can help prepare for future pandemics and other calamities by improving readiness and response skills [5]. Ultimately, the state of preparedness for an emergency on a local, state, and national level depends on first responders. During an emergency, healthcare professionals need to be properly trained and equipped [6]. Human resource development (HRD) entails a variety of areas for practitioners, such as relevant preparation, opportunities to practice new skills, drills, and exercises, and an assessment of regional capabilities.

More than half of the world’s population experienced a disaster between 2005 and 2015, with increasing severity and casualties [7]. The use of SimEx has become one of the most popular methods of training health care and allied professionals to manage disasters and emergencies in recent years [8]. It provides a safe and familiar environment for learning, allowing repetition to facilitate learning. SimEx provides healthcare professionals with the opportunity to test their skills, develop effective strategies, and receive immediate feedback. SimEx enables disaster management teams to improve their decision-making capabilities in a friendly, cooperative atmosphere [9].

Reviewing the literature on simulation exercises in disaster management & emergency preparedness is a complex task due to inconsistent use of labels and keywords in the literature. [10]. Using PubMed, a database of 36 journals has been created for emergency medicine, 24 for preparedness, and 24 for disaster management. Several keywords are interchangeable, and many keywords are used in other fields of medicine, resulting in false-positive results. Finding the right titles and articles on this subject is like searching for a needle in a haystack. Governments, non-governmental organizations, research institutes, and universities published peer-reviewed and gray literature articles relating to disaster management.

Further, despite the extensive use of SimEx, there has been no systematic study of its efficacy and best practices. SimEx’s current disaster preparedness status would provide valuable insight into its strengths and limitations, as well as its potential for improvement. Furthermore, this review may reveal gaps in current knowledge and suggest future research directions. A study might, for example, investigate various forms of SimEx and assess the impact of different aspects, such as stakeholder engagement and technology use.

In this study, we evaluated the current state of SimEx, the evaluation approaches of various simulation methods, and the challenges that organizations face. In this work, we also examined the various types of SimEx in disaster management internationally and determine best practices. By systematically reviewing the existing literature on SimEx in disaster management, the review aims to identify the different SimEx types that are being used internationally. Furthermore, the review aims to identify best practices for SimEx in disaster management as well as explore challenges in conducting SimEx exercises, which could ultimately improve their effectiveness.

### Research question and objectives

The initial analysis question posed was “Which simulation exercises are effective and feasible in disaster preparedness?”. Later it developed as “What are the current state of disaster preparation SimEx and their effectiveness in improving disaster response?“. This research question seeks to investigate the current state of SimEx practices in disaster preparation, including their types, usage frequency, and effectiveness. The review would seek to answer questions such as, What the key features of effective SimEx are? What types of SimEx are most commonly used? How frequently is SimEx conducted? and what impact they have on disaster response? By answering these questions, the review will provide insights into best practices for SimEx in disaster preparation and help to improve their effectiveness in reducing the disaster impact.

The primary objective of the project was to understand simulation exercises at present. The purpose of this was to evaluate various types of simulation exercises in terms of effectiveness and feasibility. A detailed discussion between the researchers helped determine secondary objectives including how far SimEx can work as an important educational tool for disaster preparedness to provide the desired outcome of enhanced field performance as well as obstacles and patterns in SimEx use.

The rest of the article was framed as follows. Section 2 provides search strategies for article inclusion and criteria and quality assessment. Section 3 covers the adopted search results and an overview of study characteristics. In Sect. 4, SimEx’s findings and future directions are discussed. Finally, Sect. 5 provides study conclusions and the scope of the present research.

## Methods

### Document search

Document search was conducted using available literature extracted from the databases of PubMed (Medline), Cumulative Index to Nursing and Allied Health Literature (CINAHL), Google Scholar, and Biomed central by applying the Medical Subject Headings (MeSH). The search keywords ‘disaster in emergency medicine, ‘catastrophe in disaster medicine’, ‘exercise in disaster medicine’, ‘emergency in exercise in disaster medicine’, ‘simulation in disaster medicine’, ‘drill in disaster medicine, and ‘emergency in simulation exercises’ were used. An increase in demand for SimEx in disaster management prompted the need for this analysis as well as the need for insight into this subject. According to the Preferred Reporting Items for Systematic Reviews and Meta-Analysis (PRISMA), guidelines specific open-ended questions were developed [11]. Table 1 presents the number of entries associated with each search keyword. The authors employed several keywords linked to disaster medicine and simulation exercises to search multiple databases and established inclusion and exclusion criteria, such as language (English), publication date (after 2014-till date), and relevance to the research issue, to choose which publications to include in their review. Several reviewers independently screened papers and assessed their quality and relevance to the research issue during the review process. The final selection of papers for the review was based on reviewer consensus and conformity to the inclusion and exclusion criteria.

**Table 1:** Number of search strings with given keywords

Keywords	Number of entries found
Disaster in emergency medicine	13,641
Catastrophe in disaster medicine	378
Exercise in disaster medicine	535
Emergency exercise in disaster medicine	377
Simulation in disaster medicine	656
Drill in disaster medicine	224
Emergency in simulation exercises	955

### Inclusion and exclusion criteria

**Fig. 1:**
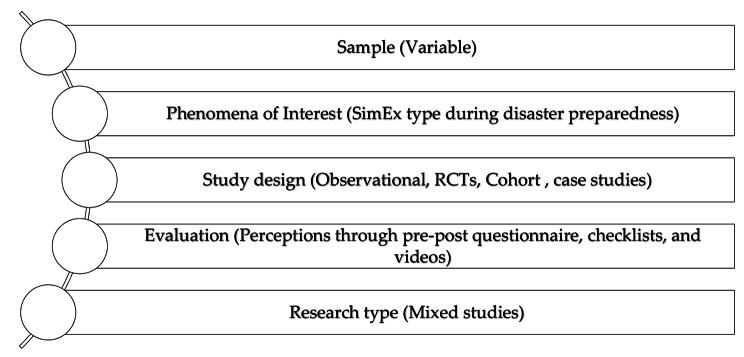
Spider strategy of study selection

Inclusion criteria included articles published in English with at least one keyword corresponding to our reviewed keywords in the title or abstract. The articles included in this study have all been peer-reviewed and published in high-quality journals. Articles published before 2014, as well as articles in languages other than English, were excluded from the study. Even though WHO guidelines were highlighted in documents before this period, they were regularly updated by the organization. Non-peer-reviewed articles were also excluded from the final search (Table 2). The process of searching and selecting studies was organized according to the SPIDER question format, which is a variation of the PICO tool. This process is illustrated in Fig. 1.

**Table 2:** Inclusion and exclusion criteria

Inclusion Criteria	Exclusion Criteria
• Articles published after 2014 • English Language articles included • Original studies, review articles, case reports, case series • Only articles published in peer-reviewed and indexed journals	• Articles published after 2014 • Articles not in the English language • Editorials, opinions, correspondences • Non peer-reviewed/non-indexed journals

### Quality and risk bias assessment

Risk of bias assessment is a critical component for conducting any form of scientific review. The current study used Newcastle-Ottawa scale (NOS) (Table 3) to determine the quality of the studies and bias risk assessment [12]. The Newcastle-Ottawa scale is a quality assessment tool that ranks the studies under review by designating stars. The higher the number of stars* is an indication of higher quality and less bias and a smaller number of stars indicates the contrary. We used a modified version of the NOS scale for this study which employed a 10-star rating system instead of a commonplace, nine-star scale. The stars measure the quality of the studies in question on key fundamental aspects i.e. (selection, comparability, and outcome). Interpretation of the NOS scale is fairly simple, the studies are rated as poor (0–4*), fair (5–6*), & good ( 7–10*).

**Table 3:** Newcastle-Ottawa scale Quality assessment form for Non-Randomized Studies included in the review

Study	1 2 3 4 5	6 7	8 9 10	Score *
Alim et al. 2015	* * *	*	* *	6
Claudius et al. 2015	* * *	* *	* * *	8
Zapko et al. 2015	* * * *	* *	*	7
Arai et al. 2017	* * * *	* *	* *	8
Fogel et al. 2018	* * * * *	* *	* * *	10
Salway et al. 2018	* * * *	* *	* * *	9
Mbanjumucyo et al. 2018	* * *	* *	* *	7
Djalali et al. 2014	* * * *	* *	* * *	9
Cramer et al. 2014	* * *	* *	* * *	8
Schulz et al. 2014	* * * *	* *	* *	8
ALuisio et al. 2016	* * *	* *	* * *	8
Johnson et al. 2017	* *	*	* *	5
Hanson et al. 2018	* * * *	* *	* * *	9
Bentley et al. 2019	* * *	* *	* *	7
Gomes et al. 2014	* *	* *	* *	6
Kilianski et al. 2014	* * * *	* *	* * *	9
Shah et al. 2013	* * * * *	* *	* *	9
Gist et al. 2016	* * *	*	* *	6
Lee et al. 2016	* * * *	* *	* *	8
Obaid et al. 2017	* * * *	* *	* *	8
Sheikhbardsiri et al. 2018	* * *	* *	* *	7
Ozella et al. 2019	* * * *	* *	* * *	9
Rüter A et al. 2016	* * * * *	*	* *	8
Burke et al. 2014	* * * *	* *	* *	8
James Le et al. 2020	* * * *	* *	* *	8
Foo Np et al. 2021	* * *	* *	* *	7
White Lewis et al. 2021	* * * *	* *	* *	8
Nejadshafiee et al. 2022	* * *	*	* *	6
Chen et al. 2019	* * * *	*	* *	7

## Results

### Search outcomes

**Fig. 2:**
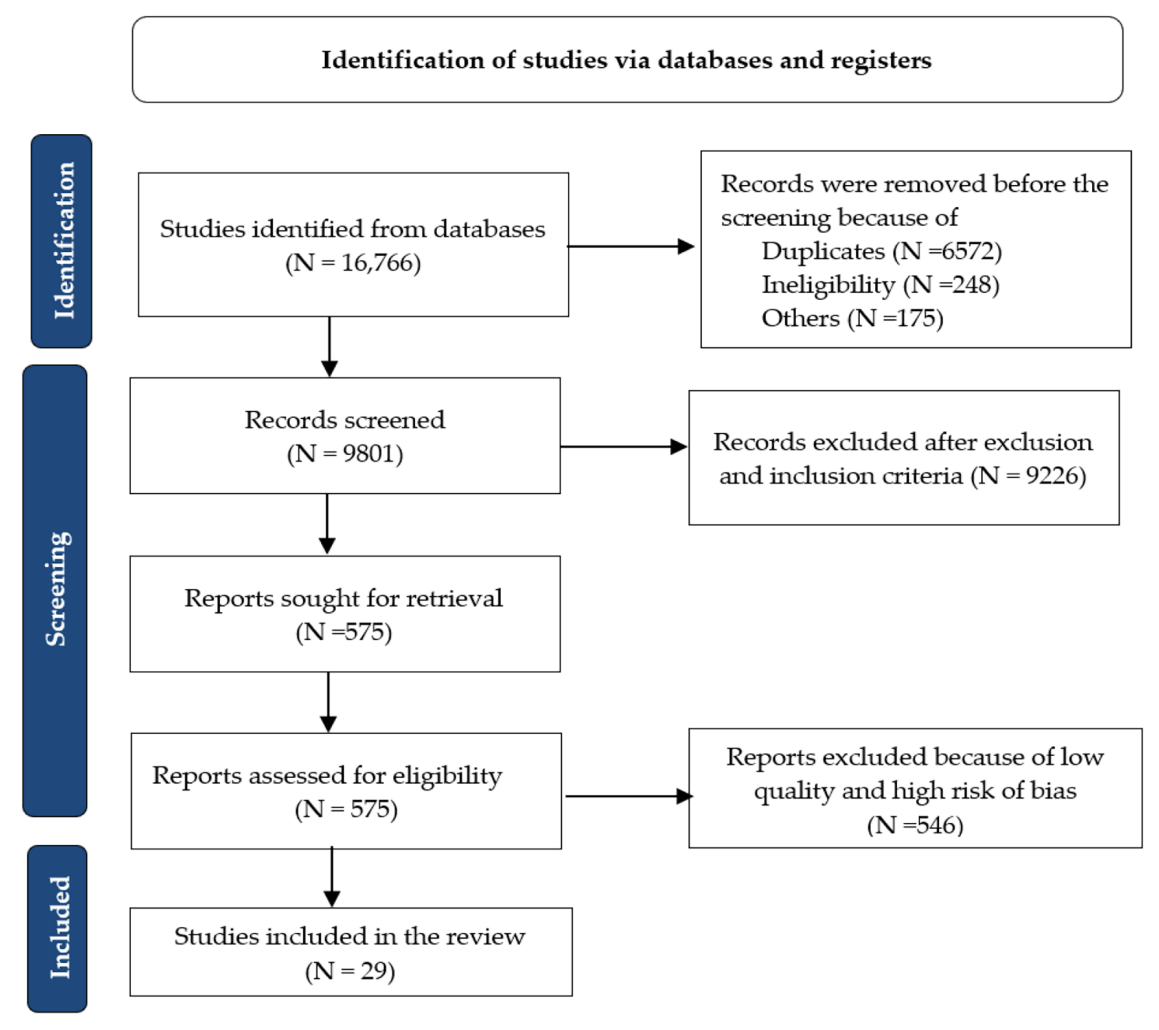
Study selection flow-chart based on PRISMA 2021 guidelines

A rigorous review of the titles and abstracts of each article was conducted by the authors to ensure that they met the inclusion and exclusion criteria. Those abstracts that lacked sufficient data were carefully assessed for significance and relevance and the search strategy was systematic based on a rational and step-by-step process. All the studies searched were included in a reference list for cataloging any relevant articles that may have been missed by the researchers at the beginning of the search. The literature search identified 16,766 papers that are associated with the given search terms. Since many search terms are used interchangeably, 6,572 duplicate works were found. Additionally, 248 records are discarded due to ineligibility because of no simulation component and focused on disaster response rather than preparation, and 175 due to other reasons such as lack of enough information on relevance or quality, and duplication. There were 9,801 articles reviewed for relevance and objectivity by the authors. Following the application of inclusion and exclusion criteria, the authors excluded 9,226 articles. Quality check criteria were applied to the remaining 575 works using the NOS scale and quality scores were independently calculated using spreadsheets. Several discussions followed, and 29 papers were selected for final review by all authors. Figure 2 presents the PRISMA flowchart for study selection.

### Study characteristics

Many studies focused on SimEx to evaluate its operational capabilities, system optimization, and staff for any emergency, including natural and man-made disasters. [13–20]. Table 4 presents the study characteristics based on SimEx type, design models, and guidelines.

#### Exercise types

Drill exercises were largely highlighted in studies [13, 21–24] followed by full-scale exercises [14, 17, 20, 24–29] and tabletop exercises [16, 18, 19]. The other type of studies reviewed included workshop discussion-based simulation exercises [13, 25, 30, 31], computer-based simulation exercise [32], and operational-based functional exercises [33–36]. The studies used different evaluation and assessment mechanisms to assess the impact and success of this simulation exercise. The studies used different evaluation and assessment mechanisms to assess SimEx’s success and impact.

#### SimEx Design models

To assess SimEx quality and effectiveness, authors need to clarify the techniques and evaluation types used. It’s because SimEx exercises and disaster drills consume an enormous amount of time and money. The following evaluation methods were most commonly used in the studies: Questionnaires/Likert scales [18, 23, 25, 29, 30, 32, 36–38] debriefing pre sessions [17, 21] and post-exercise exam [16, 37, 39]. The other evaluation methods used to assess the SimEx’s outcomes were observed for live scenarios and learning management system software such as Moodle [20], retrospective object evaluation [22], and METHANE which is a standard tool to assess and report major incident parameters include “type”, “precise location”, “hazards”, “access”, “casualty numbers”, and “emergency service [28]. To evaluate them, it is imperative to have a clear and concise strategy. All stakeholders should be able to assess the effectiveness of these exercises based on scientific theory and supported by research. Performing these evaluations can make disaster SimEx’s financially viable and attract funding for research.

An evaluation rationale should be grounded in empirical evidence, and the authors should explain which methods they will use to achieve their goals. The steps described above will lead to a logical and scientific approach to evaluating disaster SimEx and building interest in this area. According to the hospital safety index (HSI) guidelines level of preparedness of hospital staff in disasters is necessary to control the severity [14]. Competency-based evaluation tools designed for this SimEx included follow-up interviews (Cranmer et al.) and cognitive task analysis (CTA) [27], Post-exercise HSEEP participant feedback forms, and IC-specific exercise evaluation guide [35]. One assessment tool was used in some studies, while others were examined using a variety of tools including observation, interviews, proximity sensors, hot wash feedback, and electronic records [20, 26, 33, 35].

#### Guidelines development for SimEx

SimExs are developed systematically and scientifically using an exercise manual and simulation guidelines. When creating scenario-based SimExs, exercise organizers must adhere to certain guidelines for scenario development, delivery, and after-action reviews. Planning the exercise should begin with a description of the research question or learning objectives that will be addressed [13, 22]. The development of SimEx should also be carried out by professionals who examine previous research, plans, systems, and simulation designs. It is also important to develop a risk assessment plan to address the risks related to the SimEx design and participants [28]. Diverse groups of participants with defined roles assigned via different teams make exercises more dynamic and enjoyable [40]. Invitations should be based on a stakeholder analysis to determine which participants would be most appropriate for the exercise’s learning objectives [15, 37].

**Table 4:** Study characteristics

N	Study type	Country	Disciplinary	Participants	SimEx Type	Design	Guidelines		Ref
1	Original research	Indonesia	Single	309 students in the training program, 225 in a disaster drill	Discussion and operational exercises	Pre-test and post-test for in-class training, observation for disaster drill, and in-depth interview	International Council of nurses’ framework of disaster nursing competency, cross-cutting competencies for healthcare workers in disaster training, and core competencies for nurses in emergency and disaster preparedness.		(Alim et al.)
2	Original research	USA	Single	33 students (four live patients and seven computerized patients)	Operational exercises (drill)	Observation of live scenarios and learning management system software (Moodle systems; Perth, Australia) for computerized scenarios	Simple triage and rapid treatment (JumpSTART) mass casualty triage tool		(Claudius et al.)
3	Pilot study	USA	Multi	132 nursing students and 25 radiology students	Operational exercises (drill)	Debriefing session (According to the Agency for Healthcare Research and Quality AHRQ, 2011)	Jeffries simulation framework		(Zapko et al.)
4	Original research	Tokyo, Japan	Single	103 (Players:58 Simulators:22 Instructors:13 Others:10)	Operational exercises (drill)	Objective evaluation (retrospective)	A Manual was created by Tokyo universities with a scheduled time to complete tasks, necessary staff members, detailed supplies and equipment lists, and formatted documents/forms.		(Arai et al.)
5	Observational	Israel	Multi	178 healthcare workers	Operational exercises (Bio-terrorism drill)	The self-administered questionnaire was evaluated under observation according to a scoring technique of the skills competency checklist for contact precautions with the CDC	The platform was the Israeli “Orange Flame” exercise, a national preparedness buildup project conducted by the Israeli Ministry of Health per Centers for Disease Control and Prevention (CDC) guidelines		(Fogel et al.)
6	Descriptive survey	New York	Multi	62 participants, 13 healthy volunteer patients	Vertical evacuation drill. (full-scale exercise)	Self-evaluation form of 15 questions based on a Likert scale of one to five (Poor to Excellent)	Evacuation protocol was developed to review the hospital evacuation plans across the USA and to modify existing plans and protocols based on the institution’s old evacuation drills.		(Salway et al.)
7	Case report	Rwanda (Africa)	Multi	174 volunteers	Karongi (If the boat capsized), and Kanombe (If the plane crashed) exercises	METHANE is a standardized method to assess and report major incidents	Standard color-coded visual assessment triage: red, yellow, and green in major incident medical management and support (MIMMS) course		(Mbanjumucyo et al.)
8	Original research	Piedmont region of Italy	Single	61 casualties	Full-scale hospital exercise (Explosion of a gas station)	Level of disaster preparedness of hospital staff based on HIS guidelines and response performance evaluation based on the CRIMEDIM Method	Simple triage and rapid transport (START) triage		(Djalali et al.)
9	Report	Tunisia	Multi	31 participants, (19 from WHO-related missions and 12 from nongovernmental humanitarian training initiative (HTA) agencies.	Field-based exercise	Facilitators from WHO and HTI used the competency-based evaluation tool designed for this SimEx, evidence-based evaluation, Follow-up interview	The core humanitarian competencies framework developed by the consortium of British humanitarian agencies (CBHA)		(Cranmer et al.)
10	Prehospital and Disaster Medicine, Report	Germany	Multi	75 human actors, 4 four high-fidelity simulators	Prehospital mass causality incident (MCI) drill	HA and every HFS technician recorded important time points and type and number of diagnostic and therapeutic tasks on a paper and pencil questionnaire and reviewed videotapes and checklist scoring	Triage classifications according to the German association of emergency physicians		(Schulz et al.)
11	Random Control Trail	Lucknow, India	Single	60 Nurses	SimEx, and discussion-based exercises	Two standardized multiple-choice question batteries, encompassing key core content were used for assessments	American board of emergency medicine model of the clinical practice of emergency medicine with the topical focus of triage in disaster situations. Additional content was drawn from the CDC guidelines and standard emergency medicine reference texts		(Aluisio et al.)
12	Survey	Sweden	Single	13 Nurses	Small-scale computer-based simulation exercise	Pre and post-test questionnaires	Quantitative experimental method within-group design, prototype training system called Dig Emergo		(Jonson et al.)
13	Pilot	Latin America	Multi	Ministry of health representatives from 4 countries in Latin America	Workshop, table-top (Discussion-based exercise)	A combination of the Kirkpatrick model of training evaluations, pre and post-course exams, targeted activities, and a delayed participant survey enabled the Global response preparedness team (GRPT) to address 3 of the 4 levels of evaluation.	GRPT and internationalization process of the Homeland security and Exercise Evaluation Program (HSEEP)		(Hanson et al.)
14	Original research	USA	Multi	174	Operational exercise	During exercise: checklist Post-exercise: Response options on a 4-point Likert-like scale	Multi patient MCI triage Sim Wars		(Bentley et al.)
15	Case study	Brazil	Multi	26 different agencies	Full-scale exercise	CTA Techniques, direct observations, and electronic records of audio/video	External Emergency Plan (EEP)		(Gomes et al.)
16	Original research	Illinois	Multi	CCDPH staff, local and regional volunteers, and the local municipal police and fire departments	Full-scale exercise in response to a simulated anthrax bioterrorism attack	Debriefing for immediate evaluation, Observation	CCDPH has adopted the Federal Emergency Management Agency (FEMA) National preparedness cycle. Using FEMA’s HSEEP principles.		(Kilianski et al.)
17	Report	USA	Multi	36 actors/patients (medical students or emergency medicine residents)	Full-scale chlorine overexposure exercise	retrospectively evaluated electronic medical record	Kings County Hospital Center’s (KCHC’s) PICU (Pediatric Intensive Care Unit) surge plan, which was developed in conjunction with the Pediatric Disaster The coalition is a member of the New York city department of Health.		(Shah et al.)
18	Original research	USA	Multi	Emergency medicine residents, Medical Reserve Corps (MRC) volunteers	Full-scale exercise	Questionnaire	“Disaster Olympics” study design		(Gist et al.)
19	Report	Thailand	Multi	66 personnel from the Korea Disaster Relief Team, 40 medical professionals, and 106 military personnel.	3-day training, table top exercises	Videotaped, survey questionnaires, interview	Exercise co-hosted by Korea and Thailand, Third ARF DiRex (ASEAN Regional Forum Disaster Relief Exercise)		(Lee et al.)
20	Report	USA	Multi	667 participants and 83 command structures, three Medical Response Systems (MRS)	Six functional exercises	Post-exercise HSEEP participant feedback forms, IC-specific exercise evaluation guide, hot wash feedback, observation	HSEEP exercise planning guidelines were used for exercise development by the CPE exercise design team, Incident Command (IC) system framework		(Obaid et al.)
21	Report	Southeast Iran.	Multi	990 Volunteers	2-day functional exercise	checklist consisted of 13 functional dimensions based on the Iranian emergency operation plan (EOP)	The crisis management organization of the ministry of interior and the accreditation office of the Iranian ministry of health have provided guidelines and instructions for disaster		(Sheikhbardsiri et al.)
22	Original research	Italy	Multi	238 participants	Functional exercise	Observation, wearable proximity sensors	The framework of the residential course of the European Master in Disaster Medicine (EMDM)		(Ozella et al.)
23	Report	Sweden	Multi	Staff and managers of two local hospitals	Two tabletop exercises	The Hospital Incident Command System (HICS) and the Disaster Management Indicator model (DiMI)	The Emergo Train model (ETS) was used as the simulation tool		(Rüter et al.)
24	Original research	USA	Multi	In three local hospitals, staff including physicians, nurses, and nonclinical workers	Full-functional disaster exercise	Observation, Interviews, quantitative and qualitative feedback from exercise participants and observers	Mixed methods to comprehensively assess the current state of disaster preparedness, evidence-based, pediatric-specific disaster triage systems		(Burke et al.)
25	Randomized Control Trail	Haiti	Multi	480 community members	Three days of educational and training exercise	3 interviews were conducted after the intervention of baseline, 3 and 7 months post-intervention respectively	Community-based, integrated disaster preparedness randomized control trial		(James et al.)
26	Observational	Taiwan	Multi	This full scale was carried out jointly between 8 DMAT teams and 86 USAR teams	Full-Scale exercise	The researchers assigned 6 scholars from Taiwan’s society of emergency medicine and a non-government expert from Hong Kong to work as exercise evaluators and examiners. every expert was responsible for assessments at different times and in different disaster scene areas.	Full-scale exercise organized by A Nan Hospital of China Medical University and the fire bureau of Tainan City in collaboration with the city government and the Ministry of Health		(Foo et al.)
27	Clinical trial	USA	Single	31 students	Educational and training exercise in disaster preparedness for high school students.	Used a pre and post-intervention survey to determine the impact of a disaster preparedness education intervention. The tool utilized was the adapted Emergency Preparedness Information Questionnaire (EPIQ)	The experimental group was given training on bioterrorism, the START method, demonstration and return demonstration of first aid, then shock, and bleeding treatments. Practical training with spine board and cervical collar application		(White-Lewis et al.)
28	Cross-sectional	Iran	Multi	21 Specialists	A functional exercise (drill) for the possibility of nursing interventions	The nurses present at the exercise site worked in teaching hospitals in Kerman and sent the information related to hypothetical injuries to experienced nurses using the equipment (Internet) available at the exercise site and provided the target care after receiving their responses.	The operations-based exercise scenarios that included the scale of the earthquake, victims’ number, the affected area, injury type, and the local hospital capacity was done in Kerman Medical hospital.		(Nejadshafiee et al.)
29	Observational	Taiwan	Multi	40 standardized patients	Three identically designed full-scale exercises	Written in the script were all of the simulated injuries that needed to be performed on the recruited patients. To test participants’ performance, a mock wound makeup based on their scripts was applied to the simulated wound.	As part of the SimEx training, participants set up temporary field medical stations while classifying and treating simulated patients. Triage and treatment areas were separate at the field medical stations. According to the script, they performed their injuries and reacted to management instructions.		(Chen et al.)

## Discussion

The review analyzed the diverse range of SimEx exercises carried out in disaster and emergency medicine and compared the SimEx practices observed in various studies. The study also examined the challenges & obstacles to effective SimEx implementation and proposed specific recommendations to enhance disaster preparedness and response plans, processes, and systems, based on the 2017 report by the World Health Organization [41]. It is also claimed that when disaster exercise assessments are based on both quantitative and qualitative data, the evaluation conclusions are more meaningful [42]. Disaster exercises offer certain advantages in terms of convenience of use, function-driven nature, precision, consistency, validity, reliability, and cultural considerations [43, 44]. Therefore, the primary study objective of identifying and investigating patterns was achieved by a better understanding of the SimEx models that are currently being used in various disaster planning and emergency response scenarios.

In the absence of research, it is not known whether exercise assessment techniques are effective or superior. However, one evaluation method, such as video and photography, has been evaluated and found to be efficient [14, 18, 27, 38]. Video evaluations can provide some benefits, such as the ability to evaluate better, provide a secondary evaluation, and display the participant’s performance. The time saved by video debriefing can also be used for learner rehabilitation, scenario modification, or other instructional tasks. According to studies, numerous tools are available for assessing disaster recovery exercises. The inclusion of such tools is merely the responsibility of hospitals and is frequently dispensed as function-based in checklists. There is no comprehensive tool that can apply to all healthcare systems, including well-being, therapeutic interventions, and assistance [14, 17, 19, 20, 38].

In the present analysis, we included large-scale studies to understand the potentiality of SimEx approaches in disaster preparedness [14, 17, 20, 24, 26]. It is reported that choosing evaluators was an essential part of SimEx designing since they are potentially influenced by the evaluator’s perception, judgment, and knowledge of disaster management, critical care, and preparedness [17, 18, 20, 33]. Our analysis shows that the majority of SimEx exercises conducted around the world were tabletop exercises. Studies suggest that discussion-based tabletop exercises are the easiest to organize, conduct, and evaluate, especially when there are a large number of participants. Exercises based on scenarios (such as drills or full-scale exercises) require more preparation, financing, and organization [45].

Simulators, drills, and training sessions are increasingly being integrated into post-graduate medical training courses around the world. There has been a massive transformation in the medical world in terms of resources, infrastructure, technology, and public research in recent decades. Employees have been equipping themselves with the necessary skills to deal with catastrophes as part of this transformation. This has led to the creation of disaster simulation centers and the conduct of SimEx at universities and colleges. Super-specialized organizations have also emerged [46]. Further, studies by Luan D et al., Huang S et al. and Lyu et al. highlight the importance of incorporating natural hazard risk assessment and emergency response planning into infrastructure design and planning. They also showcase the potential benefits of using advanced modeling and optimization techniques to improve the performance and resilience of infrastructure systems. Overall, these articles contribute to a growing body of research aimed at promoting the safety, sustainability, and resilience of infrastructure systems in the face of natural hazards and other challenges [46–48].

The health sector is lacking evidence and information regarding the interminable implications of exercise on preparedness and response in an emergency. Participating in SimEx results in improved emergency plans that will lead to an understanding of the weaknesses and limitations of an individual or an organization. This does not guarantee that this understanding leads to actual improvement and more effective emergency management. It is difficult to demonstrate SimEx’s effectiveness at an institutional level as long as there is no evidence of any long-term positive effect on public health emergency preparedness.

Globally, there has been a lack of consistency in the response to the COVID-19 pandemic. Various countries have taken sequestered responses to worldwide problems, which makes greater preparation for pandemics, disasters, and simulations imperative. Moreover, more research is required in the area of disaster preparedness to complete the knowledge gap. There is no evidence to support the effectiveness of current exercise assessment techniques. To determine the usefulness of various forms of exercise assessment techniques in the future, emergency management experts should conduct immersive experiments. Using the current available evaluation tools and strategies, this review will contribute to improving the readiness of various sectors of the healthcare system. In this way, disaster management can be implemented successfully.

In the future, researchers and practitioners can use the current study results on SimEx in disaster preparation to improve its effectiveness in a variety of ways, including.

- *Creating standardized SimEx protocols*: Research and practitioners can use the study findings to create standardized SimEx protocols, which can ensure consistency in SimEx practices and facilitate comparison between programs.
- *Integrating more rigorous assessment*: SimEx programs may benefit from a more rigorous evaluation, which can identify areas for improvement and ensure that they are meeting their intended goals.
- *Addressing implementation barriers*: SimEx implementation may be hampered by resource constraints or stakeholder apathy. It may be possible to overcome these hurdles in the future by developing solutions to solve resource constraints or by including stakeholders early on in SimEx design.
- *Finding opportunities for improvement*: The assessment may indicate particular areas for improvement in SimEx procedures, such as the need for more realistic scenarios or greater interaction with other disaster management systems. Future studies or practices can concentrate on tackling these specific areas to increase SimEx’s efficacy.

Overall, the findings of a systematic assessment of disaster simulation exercises may be utilized to drive the development and implementation of SimEx techniques, resulting in better disaster preparedness and response.

## Conclusion

In this study, we examined current methodologies for evaluating safety interventions following accidents and disasters. The healthcare industry has conducted drills and operations to prepare for disasters and accidents. Mass casualty incidents (MCI) are characterized as overpowering events in which patients outweigh locally available resources. These events require a robust emergency response which usually necessitates support from the state or region. [49].Diverse approaches and methods should be used according to the type and purpose of the activity. Healthcare facilities may use a variety of approaches and strategies to plan safety actions and assess disaster response. Exercises can be tabletop, functional, or full-scale and are used to evaluate emergency response protocols and highlight areas for improvement. Performing post-incident evaluations to assess response activities’ efficacy and suggest improvements. Training employees to be prepared to handle crises by creating and executing training programs. Establishing alliances and collaborations with other organizations to share resources and improve response skills. It is recommended that disaster preparedness in healthcare requires a multifaceted approach. In this regard, it is essential to consider the specific needs, resources, and goals of the organization.

## Data Availability

The datasets used and/or analyzed during the current study are available from the corresponding author upon reasonable request.

## Abbreviations

CINAHL: Cumulative Index to Nursing and Allied Health Literature
CTA: Cognitive task analysis
HRD: Human resource development
HSI: Hospital safety index
MCI: Mass casualty incidents
MeSH: Medical Subject Headings
NOS: Newcastle-Ottawa Scale
PRISMA: Preferred Reporting Items for Systematic Reviews and Meta-Analyses
SimEx: Simulation exercise
WHO: World Health Organization
