# Correction: Systematic review on the current state of disaster preparation Simulation Exercises (SimEx)

**DOI:** 10.1186/s12873-023-00836-4

**Published:** 2023-06-06

**Authors:** Syed Sarosh Mahdi, Hafsa Abrar Jafri, Raheel Allana, Gopi Battineni, Mariam Khawaja, Syeda Sakina, Daniyal Agha, Kiran Rehman, Francesco Amenta

**Affiliations:** 1 Jinnah Medical and Dental College, Department of Community Dentistry, Sohail University, Karachi, Pakistan; 2 Division of Clinical Oral Health Sciences, School of Dentistry, International Medical University, Kuala Lumpur, Malaysia; 3 Department of Paediatrics & Child Health, Aga Khan University Karachi, Karachi, 74800 Pakistan; 4 Clinical Research Centre, School of Medicinal and Health Products Sciences, University of Camerino, 62032 Camerino, Italy; 5 Sociology Department, University of North Texas, Denton, TX76203 USA; 6 Division of Restorative Dentistry, International Medical University, Kuala Lumpur, Malaysia

**Correction: BMC Emerg Med 23, 52 (2023)**

**https://doi.org/10.1186/s12873-023-00824-8**

The original article [1] contained an error in author, Gopi Battineni’s name which has since been amended.
